# Artificial Intelligence in Ocular Transcriptomics: Applications of Unsupervised and Supervised Learning

**DOI:** 10.3390/cells14171315

**Published:** 2025-08-26

**Authors:** Catherine Lalman, Yimin Yang, Janice L. Walker

**Affiliations:** 1 Department of Pathology and Genomic Medicine, Thomas Jefferson University, Philadelphia, PA 19107, USA; catherine.lalman@students.jefferson.edu; 2 Sidney Kimmel Medical School, Thomas Jefferson University, Philadelphia, PA 19107, USA; 3 Department of Electrical and Computer Engineering, Western University, London, ON N6A 3K7, Canada; yimin.yang@uwo.ca; 4 Department of Ophthalmology, Thomas Jefferson University, Philadelphia, PA 19107, USA

**Keywords:** ocular transcriptomics, eye, artificial intelligence, supervised and unsupervised machine learning, retina, cornea

## Abstract

Transcriptomic profiling is a powerful tool for dissecting the cellular and molecular complexity of ocular tissues, providing insights into retinal development, corneal disease, macular degeneration, and glaucoma. With the expansion of microarray, bulk RNA sequencing (RNA-seq), and single-cell RNA-seq technologies, artificial intelligence (AI) has emerged as a key strategy for analyzing high-dimensional gene expression data. This review synthesizes AI-enabled transcriptomic studies in ophthalmology from 2019 to 2025, highlighting how supervised and unsupervised machine learning (ML) methods have advanced biomarker discovery, cell type classification, and eye development and ocular disease modeling. Here, we discuss unsupervised techniques, such as principal component analysis (PCA), t-distributed stochastic neighbor embedding (t-SNE), uniform manifold approximation and projection (UMAP), and weighted gene co-expression network analysis (WGCNA), now the standard in single-cell workflows. Supervised approaches are also discussed, including the least absolute shrinkage and selection operator (LASSO), support vector machines (SVMs), and random forests (RFs), and their utility in identifying diagnostic and prognostic markers in age-related macular degeneration (AMD), diabetic retinopathy (DR), glaucoma, keratoconus, thyroid eye disease, and posterior capsule opacification (PCO), as well as deep learning frameworks, such as variational autoencoders and neural networks that support multi-omics integration. Despite challenges in interpretability and standardization, explainable AI and multimodal approaches offer promising avenues for advancing precision ophthalmology.

## 1. Introduction

Understanding the cellular and genetic landscape of ocular tissues is fundamental to advancing knowledge of eye development, physiology, and disease. The eye is a complex organ composed of many different tissues, including the retina, the retinal pigment epithelium, the choroid, and the optic nerve in the posterior eye and the cornea, the lens, the ciliary body, the iris aqueous humor, and the trabecular meshwork in the anterior segment [1,2,3]. As the eye develops from different embryonic tissues, including ectoderm (both surface and neuroectoderm) and mesoderm, with important contributions from neural crest cells and mesenchymal cells, a complex and delicate network of genomic interactions must be precisely regulated to ensure normal morphogenesis and functional differentiation for proper visual function [1,4,5]. Given this spatial and cellular heterogeneity, transcriptomic analysis approaches, including microarrays and bulk and single-cell RNA-seq, have emerged as powerful strategies for investigating ocular biology at multiple scales. For example, bulk RNA-seq has enabled transcriptome-wide profiling across many ocular tissues and revealed age-related inflammatory genes in the choroid and dynamic changes in the Wnt, Hedgehog, and Notch pathways during early retinal progenitor cell proliferation [6,7]. More recently, single-cell RNA-seq (scRNA-seq) has generated high-resolution gene expression maps of the retina, the RPE (retinal pigment epithelium), and the choroid, enabling the identification and molecular characterization of distinct cell populations [8,9,10,11]. Transcriptomic approaches have also provided valuable insights into the molecular pathogenesis of numerous eye diseases, including keratoconus, glaucoma, thyroid eye disease (TED), AMD, and DR. Microarray transcriptomic studies have identified disease-associated gene signatures in glaucoma, TED, and DR [12,13,14]. Bulk RNA-seq has implicated dysregulation of the Wnt and Notch1 pathways in the corneal degradation observed in keratoconus [15]. Similarly, scRNA-seq analyses have revealed inflammatory and immune-related transcriptional signatures that may contribute to DR severity and AMD progression [16,17].

However, while transcriptomic analyses have significantly expanded our understanding of differential gene expression in the eye, the datasets they generate often comprise tens of thousands of genes measured across hundreds to millions of cells [18]. Furthermore, these datasets are frequently noisy and exhibit nonlinear structures, which presents challenges for conventional analytic approaches [19]. To address this, many AI algorithms, ranging from unsupervised techniques to various ML algorithms, have been developed and employed. While statistical transcriptomics focuses on detecting differentially expressed genes using predefined models, AI-based approaches uncover complex, nonlinear patterns and enable predictive analysis of high-dimensional gene expression data. These approaches have been used to cluster retinal cell types in single-cell atlases, identify ferroptosis-related gene signatures in keratoconus, and classify transcriptomic profiles in glaucoma using predictive models [8,10,20,21]. As such, applying AI algorithms to large transcriptomic datasets offers an opportunity to uncover hidden regulatory networks and identify disease-associated biomarkers more efficiently. In fact, these strategies have been successfully applied in identifying genes related to the progression of AMD, DR, and keratoconus [17,21,22,23,24].

Despite the growing number of studies applying AI to ocular transcriptomic datasets, to the best of our knowledge, there are currently no comprehensive reviews synthesizing these approaches across multiple transcriptomic modalities and disease contexts in the eye [25,26,27]. In contrast, several articles have reviewed the applications of AI to diagnosing ophthalmic diseases, summarizing numerous studies that have been conducted on the different ways AI can be utilized to clinically diagnose ocular diseases, including glaucoma, keratoconus, DR, and AMD [28,29]. The reader is referred to the following comprehensive reviews on this topic, which is beyond the current focus of this review [28,29]. Importantly, none, however, provide a unified synthesis spanning bulk RNA-seq, scRNA-seq, and microarray data, integrating both machine learning and deep learning approaches across the full spectrum of ocular diseases. By consolidating these advances into a single, structured framework, our review fills a critical gap by offering researchers a comprehensive and methodologically diverse reference on this topic. This is timely and useful considering how quickly AI-based technologies are being integrated to reshape big “omic” data analyses in the eye, providing both new opportunities and potential challenges, all of which are considered and discussed in this review. 

We conducted a structured literature search of PubMed, Scopus, and Google Scholar for articles published between 1 January 2019, and 1 August 2025. Search terms combined keywords related to transcriptomic modalities and AI methods: transcriptomics, RNA-seq, microarray, machine learning, deep learning, artificial intelligence, and ophthalmology. Boolean operators and truncation were applied to broaden results (e.g., (“RNA-seq” OR transcriptomics) AND (“machine learning” OR “deep learning”) AND ophthalmology). Eligible studies included primary research applying AI (machine learning or deep learning) to microarray, bulk RNA-seq, or single-cell RNA-seq data in ocular tissues or diseases using either human or animal data with translational relevance to ophthalmology. Review articles were also consulted for background information and to identify additional relevant primary studies. We excluded commentaries, conference abstracts, preprints, unpublished studies, non-English publications, and research not involving transcriptomic data or unrelated to ophthalmology. All titles and abstracts were screened by a single reviewer, with full texts assessed to confirm eligibility. Quality assessment included recording study design, dataset size, transcriptomic modality, AI approach, validation strategy, and reproducibility measures (e.g., code or data availability). While nearly all studies described internal validation (e.g., cross-validation, train/test splits), external validation using independent cohorts was uncommon. We distinguished between openly accessible custom pipelines (e.g., GitHub repositories or dedicated websites) and cases where authors relied on standard public libraries (e.g., scikit-learn, Seurat) or indicated that the code was “available upon request.” Only the former was classified as publicly available code.

**Figure 1:**
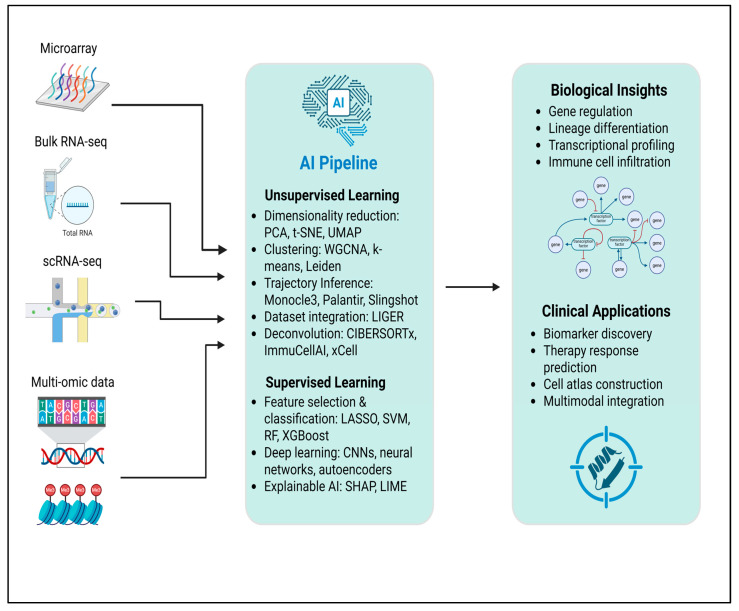
AI-assisted transcriptomic analysis pipeline and clinical applications. Transcriptomic and epigenomic data are analyzed using unsupervised and supervised machine learning approaches to uncover biological insights and inform applications like biomarker discovery, therapeutic prediction, and cell atlas construction. Created using BioRender.com. (accessed on 18 August 25, latest web-based version).

We begin by outlining transcriptomic modalities, including microarray, bulk RNA-seq, and single-cell RNA-seq, to provide context for each data structure’s advantages and analytical challenges. We then describe the AI approaches that have been previously applied, including unsupervised shallow learning, unsupervised deep learning, supervised shallow learning, and supervised deep learning. We also describe their roles in data preprocessing, pattern discovery, and predictive modeling. Finally, we synthesize these applications across several ophthalmic diseases, including corneal disorders, retinal development, macular degeneration, diabetic retinopathy, glaucoma, thyroid eye disease, and posterior capsule opacification, emphasizing the biological insights that were identified. Figure 1 summarizes these AI approaches and the associated biological clinical outputs.

## 2. Transcriptomic Modalities

### 2.1. Microarray

Microarray transcriptomic profiling, which was one of the earliest high-throughput approaches for measuring genome-wide gene expression, uses pre-designed DNA probes fixed to a chip, allowing only known transcripts to be detected [30,31]. Although microarray use has largely given way to RNA-seq technologies, which involves direct sequencing, microarrays remain valuable due to their cost-effectiveness, standardized platforms, and broad availability of archived datasets [32]. Traditional analytic pipelines for microarray data already incorporate various computational tools for normalization, such as limma and downstream pathway enrichment using DAVID or GSEA [33,34,35,36]. However, microarray datasets are sensitive to both technical and biological sources of variability, requiring robust normalization and correction to ensure comparability [37]. Variability can be introduced during sample collection, RNA extraction, labeling, hybridization, and scanning, potentially obscuring true biological differences if not carefully controlled [38,39]. The quality of the sample itself, including RNA integrity and yield, further contributes to noise and technical variance [40]. Furthermore, batch effects are a major source of confounding bias in microarray analyses and can obscure true biological differences unless addressed through statistical approaches [41]. Moreover, microarray platforms’ lower dynamic range and inability to detect novel transcripts or isoforms can constrain biological interpretation [42]. 

In recent years, AI-based methods have been applied to address these challenges by enhancing signal extraction, reducing noise through feature selection and dimensionality reduction, and integrating multi-cohort datasets to improve robustness. However, their performance ultimately depends on the quality of the underlying data, underscoring the need for rigorous normalization, batch correction, and standardized processing. For example, Suo et al. utilized limma to normalize data and correct for batch effects to integrate four different GEO microarray datasets. They then utilized SVM and RF to identify genes involved in the pathogenesis of open-angle glaucoma. Similarly, Wu et al. also used classification models, including support vector machine–recursive feature elimination (SVM-RFE), to identify ferroptosis-related genes that may play a significant role in the development of keratoconus [12,21]. As such, microarray data remain a valuable resource for hypothesis generation and validation, particularly when coupled with AI-based modeling.

### 2.2. Bulk RNA-Seq

Bulk RNA-seq was the first widely adopted high-throughput transcriptomic tool, and it has been used to analyze the gene expression patterns in mixed cell populations at scale [43]. In ophthalmology and vision research, bulk RNA-seq has been extensively applied to investigate gene expression changes that occur with keratoconus, glaucoma, cataract development, and retinal degeneration [44,45,46,47]. Traditionally, these studies have relied on established computational pipelines for read mapping and quantification, using tools like DESeq2, limma, edgeR, and BaySeq [33,48,49,50]. Differential expression analysis is then typically performed to identify statistically significant gene changes between conditions, while principal component analysis is frequently used for exploratory data visualization and dimensionality reduction [51]. Furthermore, tools like DAVID and clusterProfiler are widely used for functional enrichment analysis, enabling researchers to assign differentially expressed genes to biological processes, molecular functions, and canonical pathways, such as those in Gene Ontology (GO) or KEGG [34,52]. Although RNA-seq’s scalability and cost-effectiveness have enabled researchers to analyze a wide range of biological systems, the technique’s complex workflow presents numerous challenges that must be addressed to ensure the extraction of meaningful biological insights [53,54]. At any stage of the RNA-seq pipeline, technical or analytical issues can introduce bias into the dataset [54]. Examples include intergroup sample variability, sample to sample variation, batch effects, and the misuse of normalization methods. Tissue heterogeneity, in particular, remains a significant source of confounding variation, as differences in cellular composition can obscure true biological signals [53,55,56,57]. These sources of variation can generate spurious associations and limit the reproducibility of transcriptomic findings. Addressing them requires standardization of experimental protocols and rigorous quality control measures, such as RNA integrity assessment, normalization, batch effect correction, and computational deconvolution, to ensure data are both comparable and biologically meaningful.

While these statistical tools have proven effective in identifying enriched pathways and gene groups, they often fall short when dealing with high-dimensional, noisy datasets and subtle, nonlinear regulatory relationships. Moreover, traditional enrichment approaches frequently return thousands of significant pathways, leaving researchers with limited guidance on how to prioritize biological relevance [58]. 

AI-based approaches, including ML and DL, offer more scalable solutions by uncovering patterns not detectable through linear methods and improving feature selection in the context of thousands of correlated variables [59,60,61]. These methods enhance exploratory and predictive power in transcriptomic studies by enabling the identification of latent biological structure and robust biomarkers [62].

This shift is exemplified in ophthalmic studies. For instance, Cheng et al. used limma and DESeq2 to normalize and identify differentially expressed genes in corneal and keratoconus datasets, followed by KEGG and GO enrichment via clusterProfiler. Their workflow incorporated sample quality control through RNA integrity assessment and normalization, which helped reduce technical variation. LASSO and SVM-RFE were applied to select key diagnostic biomarkers that were integrated into a predictive nomogram [63]. Similarly, Huang et al., combining edgeR and shinyGO with supervised ML algorithms, implemented batch effect correction to mitigate site-specific biases to improve feature prioritization [20]. Wang et al. employed limma and clusterProfiler for normalization and enrichment, followed by supervised ML, and addressed tissue heterogeneity by using computational deconvolution to adjust for cell type composition [60]. These examples illustrate a broader methodological transition, as AI is no longer a supplementary tool but increasingly forms the analytical backbone for interpreting bulk RNA-seq data at scale. However, their accuracy and generalizability depend heavily on the quality and consistency of the underlying biological data. Incorporating rigorous quality control and standardization measures, such as RNA integrity assessment, normalization, batch effect correction, and computational deconvolution, ensures that these models are trained on reproducible, biologically meaningful inputs, ultimately improving predictive accuracy and interpretability.

### 2.3. scRNA-Seq

Since its first application in 2009, where Tang et al. demonstrated that transcriptome-wide mRNA profiling could be successfully performed at the resolution of a single cell, scRNA-seq has become an essential tool for unraveling cellular heterogeneity [64]. Unlike bulk RNA-seq, which provides averaged expression profiles across mixed populations, scRNA-seq enables transcriptomic profiling at single-cell resolution, revealing distinct cellular subtypes, lineage trajectories, and cell state transitions. This is especially useful for analyzing tissues in the eye, where the resolution of scRNA-seq enables fine-grained analysis of gene expression changes across diverse cell types in the retina and the RPE/choroid [9]. Although the matrices generated by scRNA-seq are information-rich, they are often unwieldy, with tens of thousands of gene features per cell and sample sizes that can reach into the millions of cells [43]. This introduces major analytical challenges, including noise, nonlinear structure, and difficulty in clustering or labeling distinct cell populations [65,66]. Furthermore, factors like donor-to-donor variability, RNA degradation, uneven cell viability, and dissociation-induced gene expression changes can alter cell type proportions and confound clustering or classification results, ultimately influencing downstream AI model performance [67,68,69]. As such, unsupervised AI techniques, particularly clustering and dimensionality reduction, including PCA, t-SNE, and Louvain or Leiden clustering algorithms, are now embedded in nearly all scRNA-seq analysis pipelines and have become standard practice for identifying structures in high-dimensional single-cell data [65,70,71,72]. Other tools, such as Seurat and Monocle3, support normalization and pseudotime analysis, while LIGER enables cross-condition and cross-species integration of single-cell datasets [10,73,74]. 

Supervised AI methods have been increasingly applied to scRNA-seq datasets in ophthalmology to both improve data processing approaches, such as cell type clustering and labeling, and also to enhance classification accuracy and biomarker identification. For example, Miao et al. introduced the SCCAF (Single-Cell Clustering Assessment Framework), a supervised learning approach that iteratively refines cluster identities by training a classifier on gene expression features, allowing for the identification of novel and previously unannotated retinal cell types [65]. Zhang et al. applied seven supervised learning algorithms to identify genes predictive of developmental stages across eight fetal retinal cell types [74]. These examples demonstrate how supervised learning enables predictive modeling and cell type inference in complex single-cell datasets, complementing unsupervised techniques by adding quantitative rigor to cell classification and gene prioritization.

## 3. Artificial Intelligence Approaches

### 3.1. Unsupervised Machine Learning

#### 3.1.1. Shallow Methods: PCA, Clustering, WGCNA

Unsupervised ML refers to computational methods that analyze and organize data without predefined labels or outcomes [75]. In transcriptomic analysis, these techniques are often used to uncover hidden patterns, group similar cells or samples, reduce dimensionality, or infer biological structure from high-dimensional gene expression data [18,43,66,76]. This is particularly valuable in transcriptomics, where each gene represents a dimension in a high-dimensional expression space [77]. Furthermore, many of the unsupervised ML methods currently utilized are considered shallow learning methods that rely on relatively simple model architectures and fewer parameters compared to deep learning [62,78]. These shallow methods are often sufficient for capturing lower-level structures in the data and widely used due to their interpretability and efficiency on smaller datasets [79]. In bulk RNA-seq ocular studies, dimensionality reduction techniques, such as PCA, are linear methods used to visualize samples with similar gene expression profiles by identifying combinations of variables that explain the greatest variance across samples [63,66,80,81,82]. Similarly, given that thousands of genes among tens of cell population clusters may be studied in a single sc-RNA-seq experiment, nonlinear dimensionality reduction techniques, such as t-SNE and UMAP, which calculate similarity scores between each pair of points within a dataset before projecting the data into a lower-dimensional space, are commonly applied [9,19,76,83]. Because PCA relies on linear projections, it is limited in its ability to capture nonlinear relationships in complex or noisy transcriptomic data [84]. While t-SNE is more effective than PCA at capturing nonlinear relationships, it often fails to preserve global structure, making it difficult to interpret trajectories or distances between clusters. In contrast, UMAP tends to better preserve both local and global structure, but its performance can be less stable on small datasets [85]. Another challenge in processing scRNA-seq data is that technical noise can be misinterpreted as true gene expression patterns. To address this, several imputation tools have been developed. One example is MAGIC, which improves data quality by constructing a graph of cells with similar expression profiles and averaging gene expression values of closely related cells in a process called diffusion to correct for outliers and smooth out noise [86,87]. Furthermore, to correct for batch effects, where gene expression levels across different samples vary systematically, unsupervised integration methods, such as Harmony, which aligns single-cell datasets across different batches or conditions by iteratively adjusting low-dimensional embeddings to minimize technical variation, are employed, as well [88,89,90].

Wang et al.’s (2022) multiome used Harmony to identify cell types, and several unsupervised clustering techniques exist, including k-means clustering, hierarchical clustering, graph-based clustering, and density-based clustering [91,92]. DEGreport, for example, utilizes hierarchical clustering, which calculates the correlation between expressions among groups of genes to better visually identify groups of genes that share similar expression profiles [93,94,95]. Wang et al. (2023) used this method to identify clusters of genes that were consistently expressed with the progression of AMD, while Wang et al. (2022) used DegReport to identify genes related to the progression of DR [16,17]. On the other hand, WGCNA is a slightly more complex gene clustering tool that constructs a co-expression network by first calculating pairwise correlation before further refining gene relationships using a topological overlap matrix (TOM) and then applying hierarchical clustering to prune connections that may not be as integral to disease states or other clinical traits [96]. In particular, WGCNA not only groups genes that demonstrate similar regulation patterns but also quantifies the relationships between the genes that are expressed within a sample. For example, Dong et al. utilized WGCNA to identify genes that may be associated with Sjogren’s syndrome to identify those that may be associated with keratoconjunctivitis sicca development [81]. WGCNA was also employed by Huang et al. and Ma et al. to identify key CD8+ T-cell-related genes in DR and to confirm that ML-prioritized genes are a part of GO pathways associated with AMD [97]. While hierarchical clustering algorithms have been applied in both bulk RNA-seq and scRNA-seq studies, graph-based clustering techniques, such as Leiden, play an integral role in scRNA-seq experiments and are used to identify cell populations that share expression profiles [65,71]. 

Another important application of unsupervised algorithms in scRNA-seq analysis is pseudotime inference, which computationally models dynamic cellular processes, such as differentiation. In this framework, cells are projected onto a trajectory graph, where each branch represents a potential lineage. The position of each cell along the trajectory defines its pseudotime, the relative measure of a cell’s progress through a biological process, inferred from gene expression patterns rather than chronological time. Many different algorithms exist, which differ in their ability to capture branching trajectories, resolve temporal resolution, handle complex topologies, and integrate prior biological knowledge or covariates into the modeling process [98]. On the other hand, Monocle3 reconstructs developmental trajectories by using UMAP to embed cells into low-dimensional space, constructing a graph that follows the shape of the data, and walking that graph to order cells along pseudotime. Palantir models cell fate as a probabilistic process, using diffusion-based graphs and Markov chains to calculate both pseudotime and the likelihood of each cell adopting a particular fate [99]. While analyzing the differentiation of retinal neuronal cells, Li et al. used a combination Monocle3 and Palantir to reconstruct their developmental trajectories [73]. 

As the previously mentioned techniques become more integrated into single-cell analysis pipelines, comprehensive toolkits, such as Seurat, which performs clustering, dimensionality reduction, trajectory inference, and batch correction using these methods, have become increasingly widespread [100,101]. For example, Jia et al. used Seurat to perform normalization, dimensionality reduction, and unsupervised clustering of their scRNA-seq and construct a cellular atlas of the trabecular meshwork in glaucomatous and healthy non-human primates, identifying 14 distinct cell types and contraction-related genes that were significantly downregulated in glaucoma [102]. Similarly, Zhang et al. and Li et al. used Seurat to perform normalization, dimensionality reduction, and clustering of macular degeneration datasets and fetal retinal transcriptomic and epigenetic data [24,73]. 

As transcriptomic studies increasingly span multiple conditions, modalities, and species, advanced integration frameworks have been developed to harmonize data while preserving biological variation. One such approach is LIGER, which uses nonnegative matrix factorization (NMF), a technique that is able to identify shared factors that capture common biological signals across all datasets and dataset-specific factors that reflect unique aspects of each dataset [103]. Liang et al. applied LIGER to integrate single-cell RNA-seq data from human, monkey, chicken, and mouse retinas, allowing for cross-species comparisons of neuronal subtypes and transcriptional regulators [10]. This enabled the identification of conserved and divergent features in retinal development and cell-type-specific expression programs. Similarly, Zhang et al. developed a Deep Subspace Nonnegative Matrix Factorization (DS-NMF) model to stratify AMD subtypes [24]. 

Among computational approaches used to interpret transcriptomic data, deconvolution has emerged as a powerful technique for estimating cell type composition within mixed tissue samples [43]. RNA-seq deconvolution is an unsupervised technique that infers the proportions of cell types within a heterogeneous sample based on gene expression profiles, which is particularly valuable when the isolation of individual cell types is challenging [104,105,106]. Among the algorithms that have been developed, CIBERSORT, which uses the ML technique support vector regression (v-SVR) to fit transcriptomic profiles against a reference gene expression matrix to profile immune cells, along with CIBERSORTx, an extension of the original algorithm that supports batch correction and provides greater cell-fraction resolution, are commonly used [107,108]. For example, Cheng et al. used CIBERSORT to calculate the proportions of immune cells in keratoconus samples [63]. Wang et al. (2023) used CIBERSORTx to estimate the infiltration of immune cells in retina tissues of different ages, while Ma et al. used the algorithm, as well as several other deconvolution techniques, to reveal the cellular composition of healthy and AMD samples [16,97]. While CIBERSORT and CIBERSORTx are designed to estimate the proportions of major immune cell types in bulk transcriptomic data, ImmucellAI has the ability to profile T-cell subpopulations and is able to recognize 18 different subtypes [109]. It was employed by Huang et al. to calculate the immune cell composition of DR and diabetic macular edema (DME) samples [61].

Gene set scoring algorithms represent another category of unsupervised transcriptomic analysis enabling functional interpretation beyond individual gene-level changes. These methods assign enrichment scores to predefined gene sets, such as Gene Ontology or KEGG pathways, on a per-sample or per-cell basis, allowing for the comparison of biological activity across experimental conditions [110,111]. One widely used method, Gene Set Variation Analysis (GSVA), transforms normalized expression data into pathway-level enrichment profiles, capturing subtle variations in pathway activity even in the absence of significant differential expression [112]. This makes GSVA particularly valuable for identifying biologically meaningful shifts in signaling or immune processes that may not be evident at the single gene level. For example, GSVA was employed by Huang et al. to quantify pathway activity related to CD8+ T-cell-associated genes in DR and applied across multiple diabetic nephropathy datasets to assess immune involvement. Similarly, a combination of GSVA and CIBERSORT was used by Wang et al. (2021) to profile the immune cell composition of RPE tissue in patients with AMD [113].

Kuchroo et al. applied an unsupervised machine learning framework based on manifold learning and diffusion-based clustering to single-nucleus RNA-seq data from AMD donor retinas. By grouping transcriptionally similar cells along a low-dimensional structure, their method reconstructed how cells transition between different states. This framework revealed distinct glial subpopulations and uncovered a conserved microglia-to-astrocyte IL-1β signaling axis driving neovascularization in late-stage AMD, demonstrating the power of geometry-aware unsupervised learning to resolve inflammatory mechanisms in retinal degeneration [23].

Put simply, this section covers tools that allow researchers to explore large gene expression datasets and uncover hidden patterns without knowing the correct answers in advance. These unsupervised tools can group similar cells, reduce the complexity of the data so that it is easier to see relationships, and reveal how cells might change over time. Some methods, like PCA, t-SNE, and UMAP, are mainly used for visualizing patterns, while others, like clustering algorithms and WGCNA, group genes or cells that behave similarly. Special approaches can also estimate what types of cells are in a tissue sample (deconvolution) or measure the activity of whole biological pathways (gene set scoring). Together, these methods give scientists a clearer picture of how genes, cells, and pathways work together in eye diseases, even when the data are messy or come from different experiments. The unsupervised machine learning techniques and their biological relevance have been summarized in Table 1.

#### 3.1.2. Deep Methods: Autoencoders, scVI

Deep learning (DL), a subset of AI built on artificial neural networks, has emerged as a powerful tool for modeling complex, high-dimensional relationships in transcriptomic data. While traditional ML relies on manual or statistical feature engineering, such as selecting subsets of genes or performing principal component analysis (PCA), deep learning models, particularly neural networks and autoencoders, can automatically learn complex, hierarchical representations of transcriptomic data directly from raw inputs [114]. Additionally, DL models are built to capture complex, nonlinear relationships in high-dimensional data and can be efficiently trained on large-scale datasets [115,116]. Because DL models contain many parameters, they generally require large datasets to avoid overfitting and learn meaningful, generalized representations when compared to the shallow learning models described earlier [79]. For example, scVI is an unsupervised deep learning framework based on variational autoencoders that models gene expression in scRNA-seq data while accounting for technical variations, such as batch effects and dropout [115,117]. Unlike traditional methods, scVI learns the probabilistic structure of the data, representing each cell and gene expression value as a distribution rather than a fixed point. This allows it to perform denoising, batch correction, dimensionality reduction, and differential expression analysis with improved robustness and biological resolution [115]. This was utilized by Liang et al., where scVI integrated over 2 million single-nucleus RNA-seq and ATAC-seq profiles. scVI enabled rigorous batch correction and clustering, which supported the identification of 110 distinct retinal cell types and subtypes, including rare populations that were previously uncharacterized [10].

In simpler terms, deep learning uses layered networks of computational neurons to automatically find patterns in large and complex gene expression datasets. Unlike traditional methods, which often require researchers to choose specific genes or features ahead of time, deep learning can learn these patterns directly from the data. This makes it especially powerful for large studies, where it can uncover subtle relationships, correct technical noise, and reveal rare cell types that might otherwise be missed.

### 3.2. Supervised Learning

#### 3.2.1. Shallow Methods: SVM, RF, LASSO

While unsupervised machine learning plays a fundamental role in many aspects of bulk RNA-seq and scRNA-seq studies, supervised machine learning, which relies on known sample labels, such as disease vs. control, or cell type identity, to classify samples or predict outcomes, has just begun to be incorporated into experimental pipelines. Like unsupervised methods, supervised techniques can be broadly categorized into shallow and deep learning approaches. Shallow learning models, such as support vector machines or random forests, are generally more interpretable and well-suited to smaller datasets [79,118]. In contrast, deep learning models, such as neural networks, are capable of modeling more complex, nonlinear relationships in large-scale data but typically require more training data and computational resources [62,78]. Supervised approaches are employed in ophthalmology to improve feature selection, predict disease progression, and model cellular development [24,60,80,97]. A variety of supervised methods have been employed in ocular transcriptomic studies, each offering distinct advantages in terms of interpretability, scalability, or modeling complexity. An overview of the shallow supervised algorithms discussed and their properties is provided in Table 2.

This table categorizes widely used supervised ML algorithms by type and describes their primary functions, strengths, and limitations in the context of gene expression studies. It highlights their applicability to high-dimensional biological data, particularly in single-cell and bulk transcriptomic analyses.

Several tools complement classifier-based approaches by enabling biological network analysis and module detection. For instance, CytoHubba and MCODE, both Cytoscape plugins, analyze protein–protein interaction (PPI) networks to reveal regulatory architecture. CytoHubba ranks genes based on topological features, such as connectedness and centrality, to identify hub genes, while MCODE detects densely connected modules that often correspond to molecular complexes or functionally related gene clusters [144,145]. These network-based methods provide complementary biological insights that may be missed by expression-based or ML-driven gene selection alone [59,60].

Miao et al. developed a clustering refinement framework for scRNA-seq data that combines simulation and supervised learning. They first simulated scRNA-seq data using multivariate normal distributions and Splatter to benchmark clustering accuracy. Their pipeline begins with over-clustering via standard algorithms, such as Louvain or Leiden, followed by supervised logistic regression to classify each cluster against all others. Marker genes were selected based on model coefficients, and poorly separable clusters were iteratively merged until all groups were distinguishable with high accuracy. Applied to the Shekhar et al. mouse retina dataset, the refined clustering closely matched the original annotations (Rand index > 0.99) and achieved an average Rand index > 0.94 on a retinal bipolar neuron dataset [65].

For keratoconus, Cheng et al. applied LASSO and SVM-RFE to select six genes from transcriptomic data, which were used to train logistic regression, support vector machine (SVM), and naïve Bayes models, all of which achieved high classification accuracy (AUCs (Areas Under the Curve) of 0.95 to 0.98) in distinguishing keratoconus from healthy controls [63]. Wu et al. similarly used SVM and SVM-RFE on microarray profiles to identify ferroptosis-related genes with strong diagnostic performance, suggesting a mechanistic link between ferroptosis and keratoconus [21]. Liu et al. analyzed keratoconus corneal data to identify genes related to programmed cell death, using LASSO and RF to define five signature genes related to ferroptosis [13]. Finally, Dong et al. performed bioinformatics and machine learning analysis on transcriptomic data from patients with Sjögren’s syndrome mediated keratoconjunctivitis sicca (KCS). They used LASSO and SVM-RFE to identify genes most associated with the development of Sjogren’s before validating three candidate genes on a separate KCS microarray dataset. Immune infiltration analysis performed with CIBERSORT revealed that these genes correlated with several immune cell types in the ocular surface, implicating them in the inflammatory response [81].

Multiple studies have also leveraged supervised learning to identify diagnostic and pathogenic markers in AMD. Han et al. used LASSO and SVM to define a 15-gene diagnostic signature, followed by the construction of RF, SVM, XGBoost, and GLM models to build a clinical prediction tool [59]. Ma et al. applied logistic regression, RF, neural networks, and XGBoost to gene expression profiles from 453 donor retinas, identifying an 81-gene glial-enriched signature that was validated through permutation testing and integration with AMD genome-wide associated data [97]. Zhang et al. employed SVM-RFE, RF, K-nearest neighbor (KNN), and Adaboost algorithms, along with differential gene expression, to select key genes to build a diagnostic model that can effectively identify AMD from test data [24]. Wang et al. integrated transcriptomic and DNA methylation data from RPE tissues to identify epigenetically regulated genes, building random forest models from expression and methylation features. The methylation-based model demonstrated superior diagnostic performance (AUC = 0.973) compared to the expression-based model (AUC = 0.825), highlighting the potential of epigenetic features for AMD diagnosis [113]. Lastly, Oca et al. analyzed RNA-seq data from peripheral blood mononuclear cells of AMD patients prior to ranibizumab treatment. Using correlation-based feature selection and RF classification, they identified a panel of four mRNAs and one miRNA that predicted treatment response with high accuracy (AUC = 0.968), effectively distinguishing responders from non-responders before therapy initiation [146].

A growing number of transcriptomic studies have applied ML to DR, aiming to identify diagnostic biomarkers, model disease progression, uncover immune and vascular mechanisms, and predict therapeutic targets across both bulk and single-cell datasets. Toh et al. developed a transcriptomic clock based on retinal vasculature gene expression in Nile rats to predict early vascular changes in prodromal DR. Using segmented regression, they identified genes associated with acellular capillary density and trained a random forest regression model on the top 14 predictors [147]. Huang et al. used LASSO and SVM-RFE in conjunction with CytoHubba and WGCNA to identify eight CD8^+^ T-cell-related genes. This diagnostic model was then validated in a separate proliferative DR fibrovascular membrane dataset and in a diabetic nephropathy dataset, which showed that the genes could also be used to distinguish early vs. late state diabetic nephropathy [60]. In a separate study, Huang et al. also applied LASSO, SVM-RFE, and CytoHubba to identify six Th17-related genes from NPDR and DME datasets. These genes distinguished DME from NPDR and were similarly validated in independent diabetic nephropathy cohorts [61]. Liu et al. used LASSO and RF algorithms to select five ferroptosis-related genes, which were validated using an external dataset. Immune infiltration profiling performed using CIBERSORT suggested that these genes were linked to altered immune microenvironments in DR [13]. Furthermore, Laich et al. used single-cell RNA and imaging mass spectrometry to study epiretinal membranes from patients with proliferative vitreoretinopathy (PVR), a fibrotic complication of advanced DR. To characterize immune and glial cell populations, they applied xCell, a supervised deconvolution algorithm that uses predefined gene signatures to infer the enrichment of specific cell types. Their analysis revealed diverse immune and glial subpopulations, including activated Müller glia and astrocytes, and showed enrichment of extracellular matrix remodeling pathways [148].

The vertebrate retina is a highly specialized neural tissue composed of diverse neuronal and glial cell types that form through tightly regulated developmental programs. Understanding these trajectories and how they vary across species, development, and disease has been significantly advanced by transcriptomic profiling, particularly at the single cell level [6,9]. However, the complexity of retinal lineage commitment and functional heterogeneity pose challenges for traditional analytic approaches [58]. A growing number of transcriptomic studies have leveraged machine learning to map developmental trajectories, classify cell types, and uncover regulatory programs in the retina. For example, Zhang et al. integrated multi-omics data using LASSO and a suite of algorithms, including AdaBoost, CatBoost, ExtraTrees, LightGBM, RF, and XGBoost, to rank features by importance to identify stage-specific markers across retinal cell types. Their incremental feature selection approach highlighted dynamic expression patterns for each type of cell in the three stages to better understand the development route of the fetal retina and the stage-specific markers [74]. Liang et al. created a multi-omics retina atlas by integrating scRNA-seq and scATAC-seq data across 69 human neuronal types and used RF classifiers to align cell identities between human and macaque retina, highlighting conserved photoreceptor profiles and divergent inner retinal neurons [10]. Lukowski et al. employed scGPS, a supervised probabilistic classifier, to compare postmortem human retinal cells with human induced pluripotent stem cell (hiPSC)-derived cones, using canonical correlation analysis (CCA) to correct donor effects and resolve 16 retinal cell types, including two rod subpopulations [8]. Li et al. combined scRNA-seq and scATAC-seq with regulatory network inference tools, such as GRNBoost2, to identify key transcription factors that govern fate transitions between retinal progenitors and Müller glia [73]. Goetz et al. integrated electrophysiology, morphology, and transcriptomics to classify 42 functional retinal ganglion cell types, training supervised classifiers on peristimulus time histograms and assigning transcriptomic identities with XGBoost [149]. Similarly, Norrie et al. used logistic regression, SVM, and RF models to map euchromatin and heterochromatin domains across the genome and integrated these maps with chromatin compartments identified through Hi-C (High-throughput Chromosome Conformation Capture) [80].

Several transcriptomic studies have utilized artificial intelligence to elucidate the molecular mechanisms underlying primary open-angle glaucoma (POAG). Suo et al. analyzed four GEO microarray datasets from trabecular meshwork and optic nerve tissues, applying batch correction, differential expression analysis, and supervised learning models (RF and SVM) to identify five diagnostic genes (AUCs 0.74–0.83). To assess the immune microenvironment, they used CIBERSORT, finding that KRT14 was positively correlated with plasma cells and neutrophils but negatively correlated with regulatory T-cells, which were increased in POAG tissues [12]. Similarly, Wang et al. used WGCNA, RF, and SVM on two microarray datasets to identify three POAG biomarkers. Immune profiling via ConsensusClusterPlus revealed distinct immune subtypes, suggesting a regulatory role of the immune system in disease progression [20]. In a study by Zhao et al., optic nerve head tissue was analyzed to identify diagnostic biomarkers of POAG. LASSO and SVM-RFE were used to select three genes, each of which showed strong diagnostic performance (AUCs 0.89–0.97). To explore regulatory mechanisms, the authors constructed a competing endogenous RNA network and a compound–mRNA interaction map before applying Mendelian Randomization, a genetic causal inference method that uses SNPs as instrumental variables, finding that DNA methylation GrimAge acceleration, an epigenetic measure of biological aging, was causally linked to glaucoma [150]. 

AI-assisted transcriptomic analysis has also been applied to thyroid eye disease (TED). Shu et al. analyzed lacrimal gland gene expression data using differential expression analysis and WGCNA to identify disease-relevant secretory genes. LASSO, RF, SVM-RFE, and XGBoost were then used to prioritize two diagnostic markers. Immune deconvolution with CIBERSORT revealed that KIAA0319 was positively associated with CD8^+^ T-cells and activated mast cells, while PRDX4 was linked to resting memory CD4^+^ T-cells [151]. In a separate study, Ma et al. used LASSO, RF, and SVM-RFE across two GEO datasets to identify six autophagy-related genes as diagnostic features. These genes were significantly correlated with immune infiltration in orbital tissues, reinforcing the role of both secretory dysfunction and immune modulation in TED pathogenesis [14].

This section describes supervised machine learning methods, which work by learning from labeled examples, such as whether a sample comes from a healthy or diseased eye, to make predictions or classify new data. Shallow models like support vector machines, random forests, and LASSO are easier to interpret and perform well on smaller datasets, while more complex models can capture intricate patterns in larger datasets but require more computational power. In ocular transcriptomics, these methods have been used to identify genes linked to diseases, predict how a disease might progress, and even forecast treatment responses. They can also highlight which genes or pathways are most important for distinguishing different conditions, giving researchers valuable clues about disease mechanisms and potential targets for therapy.

#### 3.2.2. Deep Methods: Neural Networks

Supervised DL models, such as convolutional neural networks (CNNs), have been increasingly applied to transcriptomic data to perform classification, regression, and feature prioritization tasks [152]. Like their unsupervised counterparts, supervised deep learning models require extensive tuning and large datasets; however, when trained on labeled data, they can achieve high predictive accuracy and reveal biologically meaningful relationships [47,78]. Another challenge in deep learning is its limited interpretability; while neural networks can achieve high predictive accuracy, they do not inherently reveal how specific input genes influence the model’s decisions, which can, in turn, make biological interpretation difficult [153,154]. To address this, explainable AI methods, such as SHAP (SHapley Additive exPlanations) and LIME (Local Interpretable Model–Agnostic Explanations), which can be applied across model types, are increasingly used to attribute predictions to individual genes or pathways, helping to interpret deep models in a biologically meaningful way [155,156]. These deep learning approaches have begun to gain traction in ocular transcriptomic studies, where they are increasingly applied to uncover gene expression patterns, identify disease-associated cell types, and integrate multi-omics data across diverse eye tissues and disease states. For example, Wang et al. generated scRNA-seq and scATAC-seq libraries from postmortem adult human retina and integrated them with HiChIP, which maps histone-mark-mediated chromatin interactions, and eQTL data, which link genetic variants to gene expression. To prioritize functional SNP–gene interactions, they applied convolutional neural networks (CNNs) based on the BPNet architecture to model chromatin accessibility [88,155,156].

Furthermore, in Ma et al., a neural network classifier was evaluated alongside logistic regression, RF, and XGBoost on bulk retinal transcriptomes. The inclusion of a neural network highlights the exploration of hierarchical, nonlinear modeling, although detailed architecture and performance specifics were not highlighted. However, to interpret model predictions and identify key genes driving classification, the authors applied SHAP, which attributed importance scores to individual genes and enabled the selection of an 81-gene signature enriched in glial cell types, such as microglia and astrocytes [97].

Deep learning models, such as neural networks, can learn complex patterns in large, labeled gene expression datasets to make accurate predictions about disease or cell type. While these models can be powerful, they are often harder to interpret because they do not directly show how each gene contributes to their decisions. New explainable AI tools, like SHAP and LIME, help address this by revealing which genes or pathways most influence the model’s predictions. In eye research, these approaches have been used to link genetic changes to functional effects, highlight key cell types involved in disease, and integrate multiple layers of molecular data.

## 4. AI Applications in Ocular Diseases and Retinal Development

Artificial intelligence has been increasingly applied to transcriptomic data across a wide spectrum of ocular diseases to uncover pathogenic mechanisms, identify diagnostic and prognostic biomarkers, and support therapeutic development [10,13,21,146]. These approaches span bulk, single-cell, and spatial transcriptomic modalities and integrate machine learning and deep learning frameworks for data interpretation [8,60,151]. A comprehensive summary of AI-enabled transcriptomic studies across ocular diseases, including transcriptomic modality, AI methods, and key findings, is provided in Table S1 AI-Guided Transcriptomic Studies Across Ocular Diseases.

### 4.1. Corneal Disease

Corneal diseases, such as keratoconus and KCS (keratoconjunctivitis sicca), involve multifactorial pathogenesis, including oxidative stress, immune dysregulation, and ECM remodeling. While conventional transcriptomic analyses have identified candidate pathways, recent AI-enabled approaches have enhanced our ability to disentangle complex molecular interactions and prioritize disease-relevant biomarkers.

Oxidative stress and ECM remodeling have emerged as key drivers in keratoconus. Cheng et al. analyzed bulk RNA-seq data using curated oxidative stress and ECM gene sets, integrating immune cell deconvolution via CIBERSORT and machine-learning-based feature selection. Their results identified 17 dysregulated genes that implicated innate immune activation and structural dysregulation in disease pathology. These genes, in turn, were proposed as a predictive gene signature for keratoconus, creating a nomogram for KC prediction, which was supported by expression signatures validated in patient samples and soft PDMS (polydimethylsiloxane)-cultured corneal cells [63]. Liu et al. extended this analysis by intersecting keratoconus transcriptomes with multiple cell death pathways, including ferroptosis and autophagy. Their integrated machine learning pipeline revealed hub genes enriched for TNF and IL-17 signaling, linking programmed cell death to inflammatory remodeling in the cornea [157]. Furthermore, Wu et al. investigated ferroptosis in KC using WGCNA and SVM-RFE, identifying hub genes associated with oxidative stress, immune regulation, and metal ion transport. They identified miR-184 as a potential regulatory factor, which has been previously reported to be the most abundant miRNA in corneal and lens epithelial cells, linked to horizontal protein alignment in the cornea. In that study, decreased AKR1C3 expression was found to reduce miR-184 synthesis in keratoconus, and four predicted AKR1C3-targeting drugs (indomethacin, daunorubicin, doxorubicin, and docetaxel) were highlighted as potential genetic-level interventions. No clinical validation was performed [21]. Finally, Cai et al. identified nine characteristic genes involved in the pathogenesis of keratoconus using machine learning algorithms. These genes were associated with oxidative stress, ferroptosis, inflammatory responses, and mitochondrial apoptotic pathways. Validation with a single-cell RNA-seq dataset highlighted ACSL4, an enzyme that activates polyunsaturated fatty acids and promotes ferroptosis, as the most significant hub gene, which was further confirmed by RT-PCR and Western blotting in corneal stromal cells. Notably, ACSL4 expression was significantly upregulated under conditions of reduced substrate stiffness, implicating this gene, and mitochondria-related pathways more broadly, as critical drivers in the development of keratoconus. However, these findings remain preclinical, as no clinical validation has been performed [22].

Immune involvement has also been highlighted in KCS. Dong et al. applied ML-based gene selection to a microarray dataset and identified biomarkers correlated with inflammatory cytokines in a rat corneal injury model. Upregulation of key mediators, such as JAK1, SKI, and ZBTB16, paralleled increased IL-6, IL-1β, and TNF-α expression, implicating innate immune activation in dry eye pathogenesis. While these results are based on a rat corneal injury model and require confirmation in human studies, they suggest that JAK1, SKI, and ZBTB16 could represent candidate biomarkers for future diagnostic assays or targets for immunomodulatory therapy in dry eye [81].

Together, these studies demonstrate how AI-guided transcriptomic profiling can uncover convergent biological themes across corneal diseases, including oxidative stress, immune infiltration, and ECM (extracellular matrix) disorganization, while enabling the development of predictive gene signatures for future diagnostic and therapeutic applications.

### 4.2. Acute Macular Degeneration

AMD is a complex, multifactorial disease involving aging, genetic susceptibility, inflammation, and environmental exposures. While transcriptomics has advanced our understanding of AMD’s pathogenesis, the integration of AI, particularly supervised machine learning, has enabled deeper analysis of high-dimensional gene expression data. These approaches can uncover hidden molecular patterns, identify diagnostic and prognostic biomarkers, and stratify patients into biologically meaningful subgroups. In AMD, AI-guided transcriptomic studies have revealed novel insights into immune dysregulation, glial remodeling, mitochondrial dysfunction, and aging-related transcriptional reprogramming.

Several studies have revealed immune activation as a central feature of AMD pathogenesis. Han et al. screened transcriptomic data from human donor RPE choroid tissues to identify 15 disease signature genes and constructed a five-gene clinical prediction model. They implicated several immune functions, such as macrophage activation and CD4+ memory T-cell function, as predictors, which they were able to use to differentiate molecular subtypes of AMD and predict disease progression. Although the model shows strong predictive performance in silico, its clinical utility remains to be validated in prospective patient cohorts [59]. Wang et al. further investigated immune subtype regulation in aging human retinas through immune deconvolution and methylation–transcriptome integration, showing that genes like SMAD2 and NGFR were able to predict AMD progression and were associated with specific immune system functions, including inflammatory signaling. These genes, in turn, could be potential therapeutic targets of AMD; however, as no clinical validation was performed in that study, future studies should pursue further biological testing [113]. Another study by Wang et al. focused on aging found increased infiltration of M2 macrophages and activated T-cells in association with disease severity. Using CIBERSORTx and scRNA-seq reference data, they showed that immune dysregulation was concentrated in glial cell populations, such as Müller glia and microglia. Eight age-related MGS genes were identified in these processes, potentially playing critical roles in the progression of AMD with age. While no clinical trial validation was performed, the findings suggest potential mechanisms and targets for slowing age-related AMD progression [16]. Oca et al. contributed to this immunologic perspective by identifying a blood-based transcriptomic signature predictive of anti-VEGF (vascular endothelial growth factor) treatment response, suggesting that systemic immune alterations may also modulate therapeutic outcomes. The signature, derived from PBMCs of AMD patients, comprised four mRNAs and one miRNA and retrospectively predicted a successful response to ranibizumab with good accuracy. The authors proposed that machine learning classifiers based on mRNA and miRNA profiles, particularly when combined with baseline clinical characteristics, could improve the identification of patients unlikely to respond adequately to ranibizumab and enable patient-specific treatment planning from the first visit. The study was conducted in clinical cohorts [146]. Collectively, these studies underscore the centrality of immune dysfunction in AMD’s pathobiology.

Glial cells, particularly Müller glia, astrocytes, and microglia, have emerged as consistent cellular contributors across transcriptomic studies. Ma et al. identified an 81-gene signature enriched in glial markers that predicted AMD status and overlapped with known AMD gene loci, reinforcing the genetic basis of glial involvement They also discovered a novel AMD-associated variant, rs4133124 at *PLCG2*, suggesting that genes involved in retinal glial function may drive AMD’s pathology and that disease progression may not follow a strictly linear course [97]. Kuchroo et al. used single-nucleus RNA-seq and a topology-aware clustering algorithm (CATCH) to resolve glial subpopulations across disease stages [23]. They revealed early activation states involving phagocytosis and lysosomal remodeling, and, in advanced disease, they identified a conserved microglia-to-astrocyte IL-1β signaling axis that drives angiogenesis, functionally validated in vitro and in vivo. Given that anti-VEGF therapy remains the only approved intervention for AMD and is primarily effective in advanced stages, the authors proposed that inhibiting microglia-derived IL-1β could offer therapeutic benefit by preventing further neovascularization in advanced patients or even forestalling its onset in earlier stages. These experiments were performed in vitro and in mouse models, with no clinical trials conducted to date. These studies collectively highlight glial remodeling as both an early marker and late-stage driver of AMD’s progression.

Mitochondrial dysfunction and oxidative stress are also increasingly recognized as drivers of retinal degeneration in AMD. Zhang et al. integrated bulk and single-cell RNA-seq to define two molecular subtypes based on mitochondrial gene expression profiles. They constructed and validated a 13-gene diagnostic model linked to immune microenvironment shifts, suggesting that mitochondrial stress may both result from and exacerbate chronic inflammation. Using four machine learning methods, they further identified pathways associated with these subtypes and predicted ten potential small-molecule therapeutics for AMD. While these findings provide a theoretical basis for targeted treatment, no clinical validation has yet been performed [24]. This aligns with immune-focused studies and highlights how mitochondrial and inflammatory processes intersect in AMD pathogenesis.

Additionally, Wang et al. identified 26 age-associated genes whose expression levels correlated with both chronological age and clinical disease severity. These genes were enriched in glial cell types, particularly Müller glia, microglia, and astrocytes. Bulk RNA-seq deconvolution further confirmed an increase in glial proportions with age, especially in advanced AMD. Notably, age-related immune changes overlapped with those identified in inflammatory and mitochondrial studies, suggesting a shared molecular axis of degeneration. Together, these findings support the view that aging-associated transcriptional reprogramming, glial expansion, and immune activation converge to create a permissive environment for retinal degeneration [16].

### 4.3. Retinal Development

Recent applications of artificial intelligence in retinal development have illuminated the cellular and molecular complexity of neurogenesis, chromatin dynamics, and disease-associated transcriptional programs. By integrating single-cell multi-omics technologies with ML and DL models, researchers have begun to map the regulatory architecture underlying retinal cell type specification, fate commitment, and degeneration. These studies have not only refined our understanding of normal retinogenesis but also identified critical pathways and biomarkers relevant to conditions like glaucoma, macular degeneration, and photoreceptor loss.

Multimodal and AI-based profiling has enhanced our understanding of functional diversity within retinal cell types. Goetz et al. used electrophysiological, morphological, and transcriptomic profiling of mouse retinal ganglion cells (RGCs) to define functional subtypes. Machine learning classifiers trained on light-evoked responses revealed that Tusc5 expression marked a specific RGC type with transient responses and compact dendritic fields, linking transcriptional identity to visual function and offering insights into how dysfunction in specific RGC subtypes may contribute to retinal neurodegeneration. Furthermore, the study enabled the identification of each RGC type along with its projection and wiring patterns in the brain and within the retina, as well as the underlying molecular determinants. No clinical validation was performed [149].

AI-guided transcriptomics has also deepened insight into the transcriptional regulators that govern retinal development. Li et al. integrated embryonic human scRNA-seq and scATAC-seq data to identify transcription factors, such as REST, IRX1/2, ONECUTs, and LHX3/4, that direct Müller glia and retinal neuron differentiation. Of particular note, a glial subtype (MGC2) appeared essential for supporting macular neuron formation, implicating early glial dysfunction in congenital retinal disorders. In parallel, they examined the top 25 disease-related genes across congenital and other ocular diseases, noting that elevated PI3K family gene expression was linked to retinoblastoma, mutations in mGluR6 cascade members represented the third most common cause of complete congenital stationary night blindness, and MGC2 showed the strongest associations with pathogenic genes implicated in AMD, DR, and common uveitis. The study was based on a small number of human embryonic eye samples, and clinical validation is required to confirm these findings [73]. Similarly, Zhang et al. used seven supervised ML models to prioritize stage-specific regulators across major human retinal cell types. Key biomarkers—including RELN, DAB1, ANK3, RIMS2, PDE6H, NFIA, and WIF1—were implicated in neural migration, synaptic stability, phototransduction, and retinal repair, with specific genes, such as RELN, DAB1, and RIMS2, linked to lineage specification in amacrine, bipolar, and photoreceptor cells. These findings provide a mechanistic basis for understanding developmental disorders and inherited retinal dystrophies and identifying potential therapeutic targets for retinal diseases, including cone-rod dystrophy, diabetic retinopathy, AMD, and glaucoma. No clinical trials or in vivo validation have yet been conducted [74]. Finally, Lukowski et al. created a single-cell transcriptomic atlas from postmortem human retinas, identifying 18 distinct neural retinal cell populations using unsupervised clustering and the scGPS machine learning framework. Their analysis revealed postmortem-dependent downregulation of MALAT1 in rod photoreceptors, suggesting its potential as a target to enhance photoreceptor survival and preserve retinal function. The atlas also serves as a benchmark for assessing stem-cell-derived retinal cell types and detecting early molecular changes in retinal disease. Due to the limited number of donor and profiled cells, no clinical validation has yet been performed [8].

Genomic and epigenomic integration has clarified how noncoding variants shape disease risk. Wang et al. generated scRNA-seq and scATAC-seq libraries from postmortem adult human retinas and combined them with HiChIP and eQTL data to identify functional SNP–gene interactions. CNNs based on the BPNet architecture modeled chromatin’s accessibility, prioritizing variants like rs7727244 and rs4102217 as risk loci for myopia and glaucoma. Notably, rs1532278 was shown to regulate CLU expression specifically in Müller glia, highlighting the cell-type-specific impact of noncoding variants in diseases like AMD and glaucoma. The study also nominated additional pathogenic SNP–target gene interactions (e.g., rs1874459) relevant to AMD, glaucoma, DR, myopia, and type 2 macular telangiectasia, providing a valuable resource for interpreting noncoding variation in the eye. No clinical trials have been performed [88]. 

DL has also enabled new frameworks for regenerative therapy evaluation. Schaub et al. used neural networks trained on quantitative brightfield absorbance microscopy (QBAM) images of iPSC-derived RPE cells to predict VEGF secretion and transepithelial resistance, both of which are key indicators of RPE maturity and function. Complemented by ML models like MLPs, PLSR, and RF, this approach identified morphological features predictive of therapeutic quality, presenting a clinically compatible strategy for evaluating cell therapy products prior to implantation [158].

Chromatin accessibility and transcriptional regulation at the single cell level are also being mapped to establish disease baselines. Liang et al. used snRNA-seq and snATAC-seq to construct a comprehensive atlas of healthy human retina, applying the SCENIC pipeline to reveal transcription factor modules active in specific retinal cell types, providing a molecular framework for understanding normal retinal biology and detecting regulatory disruptions in degenerative diseases. While no clinical trials were performed, these findings offer a valuable resource that may inform the design of future retinal disease studies and targeted therapeutic strategies [10].

### 4.4. Diabetic Retinopathy

DR is a progressive retinal complication of diabetes, causing significant visual impairment. There are two broad categories, including the early stage of non-proliferative diabetic retinopathy (NPDR) and the advanced stage of proliferative diabetic retinopathy (PDR). An important additional category of DR is DME, the most common cause of vision loss in patients with DR. While traditionally characterized by vascular damage, AI-enabled transcriptomic analyses have expanded our understanding of DR as a multifaceted disorder involving immune dysregulation, oxidative stress, and microvascular remodeling.

Immune activation has emerged as a key driver of DR progression. In two complementary studies, Huang et al. applied network-based analysis and supervised gene selection methods to DR-related transcriptomes, identifying stage-specific immune markers. In the first study, they found that Th17 cell infiltration increased from control to NPDR to DME and identified six hub genes, CD44, CDC42, TIMP1, BMP7, RHOC, and FLT1, associated with Th17-related inflammation. This increased progressively from control to NPDR to DME, with associated hub genes largely involved in leukocyte trafficking, angiogenesis, and cytoskeletal remodeling [61]. In another study, they focused on CD8^+^ T-cells and identified eight additional genes, IKZF1, PTPRC, ITGB2, ITGAX, TLR7, LYN, CD74, and SPI1, associated with immune activation, cell adhesion, and innate immune sensing pathways. In both studies, hub gene expression was validated by GSVA and qPCR in murine models, suggesting that adaptive immune cells, particularly Th17 and CD8^+^ T-cells, play active roles in the progression to vision-threatening DR phenotypes like DME [60].

Oxidative stress and ferroptosis have also been implicated in DR. Liu et al. analyzed microarray data to identify 40 differentially expressed ferroptosis-related genes and applied LASSO and RF to prioritize five hub genes involved in antioxidant defense, iron metabolism, and autophagy regulation. These genes were linked to immune infiltration and validated by qRT-PCR in human retinal microvascular endothelial cells exposed to high glucose, suggesting that ferroptosis may contribute to immune activation and vascular injury in diabetic eyes. Molecular docking further demonstrated strong binding of glutathione to CAV1 and TLR4, highlighting a potential ferroptosis-targeting therapeutic approach. While these findings highlight ferroptosis as a potential therapeutic avenue, in vivo and clinical validation remain necessary [13].

Microvascular degeneration and remodeling were explored by Toh et al., who performed RNA-seq on retinal vascular tissue from Nile rats and trained a random forest model to predict acellular capillary density, which is a hallmark of early DR. Their 14-gene signature provided a molecular correlate for vascular dropout before clinical signs emerge and was used in a data-driven approach to identify three candidate drugs—NVP-TAE684, geldanamycin, and NVP-AUY922—that could potentially attenuate early DR by downregulating gene expression linked to acellular capillary density. These findings offer transcriptomic insight into early-stage DR pathogenesis and a framework for drug repurposing, though in vivo and clinical studies are needed to confirm their therapeutic potential [147]. In parallel, Wang et al. analyzed macular transcriptomes across DR severity stages and identified seven genes with expression patterns that tracked disease progression. Among them, CCND1 and FCGR2B were associated with increased infiltration of M2 macrophages, supporting a role for immune remodeling in late-stage DR. By controlling for factors like age and gender, they confirmed that expression of these genes, along with proportions of memory B cells, M2 macrophages, and Müller glia, increased with DR severity. These findings suggest potential molecular and cellular targets for studying the mechanisms of DR progression. The study was based solely on in silico analysis of transcriptomic data [17].

Laich et al. extended these insights by using single-cell RNA and protein profiling to study epiretinal membranes from patients with proliferative vitreoretinopathy (PVR), a fibrotic complication of advanced DR. Their work revealed diverse immune and glial subpopulations, including activated Müller glia and astrocytes, and showed enrichment of extracellular matrix remodeling pathways. Drug-matching analysis ranked aminocaproic acid, levamisole, and TOP2A inhibitors (etoposide, mitoxantrone, doxorubicin) among the top candidates, alongside daunomycin, which has already been investigated in PVR clinical trials. These findings provide targets for developing PVR diagnostics and therapeutics, with future clinical trials representing the next step [148].

Together, these studies suggest that DR progression is shaped by converging mechanisms of immune infiltration, oxidative injury, and vascular degradation. AI-assisted transcriptomic approaches have enabled the identification of stage-specific biomarkers and clarified the contributions of Th17 and CD8^+^ T-cells, ferroptosis pathways, and macrophage-driven inflammation. These insights lay the foundation for new strategies in early diagnosis, therapeutic targeting, and individualized risk stratification in DR.

### 4.5. Glaucoma

Factors that affect the pathogenesis and development of glaucoma are still incompletely understood. As such, transcriptomic analyses have increasingly been used to uncover molecular contributors to disease onset and progression. Recent studies integrating artificial intelligence (AI) have identified gene signatures pointing to roles in neurovascular signaling, vesicle transport, and metabolic stress. 

For example, Wang et al. applied ML analysis to gene expression data filtered through an immune gene set from the ImmPort database and identified three hub genes: CD40LG, MDK, and TEK. While all three genes have immune or vascular relevance, the study primarily highlights their potential roles in glaucoma through mechanisms like neurotrophic signaling and vascular stability, suggesting that immune-adjacent pathways may contribute to disease development. Using the DGIdb database, the authors further predicted small-molecule drugs targeting these genes, providing a potential therapeutic direction for POAG. Expression patterns were validated through RT-PCR in mouse ocular tissues, but no human clinical validation has yet been performed [20]. Similarly, Zhao et al. analyzed POAG-associated optic nerve head transcriptomes using ML and Mendelian Randomization and identified three hub genes: RAB8A, PRG3, and SMAD3. They also constructed a small-molecule compound–mRNA interaction network, suggesting potential pharmacologic modulators of these biomarkers. These genes implicate vesicle trafficking and inflammatory signaling in glaucomatous neurodegeneration, with SMAD3 suggesting a potential role for TGF-β-mediated fibrosis and immune activation in POAG. Expression patterns were validated through RT-PCR in mouse optic nerve head tissue, but no human clinical validation has yet been performed [150]. Finally, Suo et al. applied machine learning to POAG-associated transcriptomic data from the trabecular meshwork and optic nerve and identified five signature genes associated with disruptions in epithelial integrity, oxidative metabolism, and iron homeostasis. Using the Connectivity Map database, the authors identified five compounds, avrainvillamide-analysis-3, cytochalasin-D, NPI2358, oxymethylone, and vinorelbine, whose gene expression profiles were inversely correlated with those of POAG, suggesting the potential to mitigate or reverse the disease state. These predictions are based solely on in silico analyses, with no in vivo or clinical validation performed [12].

### 4.6. Thyroid Eye Disease

Thyroid eye disease (TED) is an autoimmune orbital disorder often associated with Graves’ disease characterized by orbital inflammation, fibroblast activation, and extracellular matrix remodeling. While its clinical manifestations include proptosis, diplopia, and vision loss, the underlying molecular mechanisms remain incompletely understood. Recent AI-guided transcriptomic studies have expanded our understanding of TED beyond broad immune activation, highlighting more nuanced contributions from secretory dysfunction, autophagy, and immune-fibroblast interactions.

Immune activation has emerged as a prominent contributor to TED’s pathogenesis. Shu et al. analyzed lacrimal gland transcriptomes using differential expression and WGCNA, followed by supervised machine learning methods, including LASSO, random forest, SVM-RFE, and XGBoost. Two hub genes, KIAA0319 and PRDX4, were identified as diagnostic markers. Immune cell deconvolution using CIBERSORT revealed that KIAA0319 expression correlated positively with CD8^+^ T-cells and activated mast cells, whereas PRDX4 was associated with resting memory CD4^+^ T-cells. These findings suggest that both cytotoxic and regulatory immune cell populations play roles in TED’s severity and orbital tissue remodeling. The same study by Shu et al. found that secretory genes involved in tear film production and mucin secretion were downregulated in TED patients, supporting clinical observations of dry eye symptoms and ocular surface instability. The involvement of CD8^+^ T-cells and mast cells further suggests that inflammation may disrupt normal glandular function. However, these results are derived entirely from computational analyses and await validation in tear fluid from clinical cohorts [151].

Autophagy-related pathways have been highlighted in additional TED studies. Ma et al. analyzed orbital tissue transcriptomes from two GEO datasets and used a combination of LASSO, RF, and SVM-RFE to identify six autophagy-associated genes with strong diagnostic potential. These genes showed significant correlations with immune cell infiltration, linking impaired autophagic flux to immune activation and tissue remodeling in the orbit. They further performed drug–gene interaction screening to identify potential compounds targeting these hub genes, suggesting avenues for therapeutic exploration. While this suggests that defective cellular recycling may exacerbate antigen presentation, inflammation, and fibroblast activation in TED, they remain unvalidated in human samples or clinical cohorts [14].

### 4.7. Posterior Capsule Opacification

The lens has also been extensively profiled using transcriptomic and multi-omics approaches, providing a foundation for AI applications [152,153,154,155,156,157,158,159,160,161,162,163,164]. Our group recently applied supervised machine learning to transcriptomic data from an ex vivo chick lens injury model to distinguish molecular signatures associated with regenerative wound healing versus fibrotic posterior capsule opacification (PCO) outcomes. Using LASSO, support vector machines, and random forests, we identified distinct gene panels linked to wound healing (e.g., HS3ST2, ID1) and fibrosis (e.g., VGLL3, CEBPD, MXRA7), with pathway analysis implicating MAPK and HIPPO signaling in divergent outcomes. While experimental validation was performed using RT-PCR, clinical validation was not performed at that time [165]. This approach illustrates how AI-guided feature selection and classification can resolve biologically meaningful signatures in a clinically relevant model of secondary cataract formation. 

## 5. Challenges and Limitations

AI-enabled transcriptomic analyses in ophthalmology are increasingly translating molecular insights into clinical applications, from diagnostic gene panels and patient stratification tools to therapeutic target discovery and drug repurposing opportunities. However, despite the growing utility of AI in ocular transcriptomics, some limitations remain. Some ocular transcriptomic studies, especially those involving rare diseases or single-cell datasets, face challenges related to limited sample sizes and high dimensionality, which may increase the risk of overfitting and reduce generalizability [118,162]. Another challenge is the inconsistency in clustering and annotation strategies across studies, which can hinder the accuracy of downstream analyses, such as deconvolution and cell type comparisons. For example, while some studies rely on standard clustering algorithms like Louvain, others implement custom pipelines using supervised classifiers, such as scGPS in Lukowski et al., feature selection frameworks in Zhang et al., or iterative refinement methods in Miao et al., making direct comparisons difficult [8,65,74]. Additionally, if findings are not followed by in vivo or in vitro functional experiments, biological interpretation is limited [166]. While many ML models demonstrate promising diagnostic or predictive performance, their limited interpretability remains a significant concern, particularly in biological and clinical settings [167]. Complex models, such as random forests, support vector machines, and deep neural networks, often function as “black boxes,” providing limited insight into how predictions are derived [168]. Explainable AI techniques, including SHAP and LIME, offer promising solutions by attributing importance scores to individual input features [169,170]. However, these tools are not yet routinely implemented in ocular transcriptomic studies [171]. Moreover, there is a risk of over-interpreting associations without orthogonal validation using proteomics, functional assays, or genetic perturbation studies [172]. Furthermore, case-control designs are prevalent, but they do not support causal inference, and external validation is often lacking [173,174,175]. For example, while Toh et al. performed text mining to indirectly validate the three compounds that were explained to be able to inhibit the genes that composed their acellular capillary density genomic signature, no experimental studies were performed to validate their findings [147]. This is also acknowledged in both Huang et al. (2022a) and Huang et al. (2022b), where no in vitro or in vivo confirmatory studies were performed [60,61]. Furthermore, these studies mention that because the original dataset utilized came from case-control studies it is impossible to clarify the causal relationship between the expression of biomarker genes and the presence of immune cells [60,61].

Another notable limitation across the reviewed literature was the lack of publicly available code and limited external validation, underscoring ongoing challenges for reproducibility and generalizability in AI-driven transcriptomic studies. While several studies mentioned code availability, closer inspection revealed that most were not publicly accessible in practice. For example, Wang et al. (2022) stated that analysis scripts could be provided “upon request,” which we classified as not openly available [17]. Goetz et al. created an open online resource (rgctypes.org) for their retinal ganglion cell classification framework, which provides access to data and algorithms, although this represents a data portal rather than a fully documented analysis pipeline [149]. Norrie et al. deposited sequencing data in GEO and described the use of existing open-source software but did not release new custom scripts [80]. In contrast, Kuchroo et al. represent the only example in which a custom pipeline was made publicly available; their CATCH Python library was deposited on GitHub with documentation and tutorials [23]. Thus, apart from this isolated case, most studies did not provide usable custom code, reflecting a broader reproducibility gap in the field.

Finally, clinical translation remains a major hurdle. Most AI-driven transcriptomic insights have not yet been incorporated into diagnostic, prognostic, or therapeutic workflows, and regulatory pathways for such tools are still in early development [176,177]. Notably, the majority of studies reviewed here lacked direct clinical validation, limiting their immediate applicability to patient care. Key barriers include the absence of robust cross-cohort validation, lack of standardized analytic pipelines, and the need to demonstrate clinical utility in prospective trials. In addition, regulatory pathways for AI-enabled molecular tools are still in their infancy, with unresolved issues related to data privacy, reproducibility, interpretability, and integration into existing electronic health records. Overcoming these challenges will be essential to bridge the gap between discovery-driven analyses and real-world clinical application. 

In addition to technical and translational challenges, the application of AI to ocular transcriptomics raises important ethical, legal, and privacy concerns. Transcriptomic datasets, particularly when linked to clinical records or genomic metadata, can contain sensitive personal information [178]. Robust de-identification, compliance with data protection frameworks, such as HIPAA (US) or GDPR (EU), and secure data storage are essential to prevent re-identification risks [179,180]. Moreover, equitable model development requires addressing biases introduced through the underrepresentation of specific populations, which can lead to disparities in diagnostic or therapeutic recommendations [181]. Legal and regulatory pathways for AI-driven molecular diagnostics are still evolving, underscoring the importance of transparency, reproducibility, and adherence to ethical guidelines when integrating these tools into clinical practice [181].

## 6. Future Directions

Integrating multimodal data (e.g., imaging, epigenomics, proteomics) remains technically and computationally complex, although it holds substantial promise for future precision ophthalmology applications [88,177]. Recent advances in multimodal AI frameworks suggest promising avenues for integrating spatial imaging and molecular data in ophthalmology. For instance, Jackson et al. developed a high-resolution, AI-derived retinal thickness map from optical coherence tomography (OCT) and associated it with transcriptomic and epigenomic profiles from spatially matched retinal tissues. Their model uncovered spatial transcriptomic signatures linked to retinal architecture and disease-prone regions, illustrating how DL imaging phenotypes can be anchored to underlying molecular biology [11]. While most DL applications in ocular transcriptomics focus on gene expression data alone, Schaub et al. demonstrated the potential of using neural networks on non-invasive imaging to predict cell function. They trained models to forecast transepithelial resistance and VEGF secretion from label-free microscopy images of iPSC-derived RPE, highlighting opportunities to integrate morphological, functional, and molecular data [25]. 

AI-driven multi-omics integration is also gaining traction [28,29,182,183,184]. Laich et al. combined scRNA-seq with imaging mass spectrometry, a technique that maps spatial distributions of metabolites and proteins within tissue sections, to characterize immune and glial heterogeneity in proliferative vitreoretinopathy (PVR), revealing ECM remodeling pathways [148]. Imaging mass spectrometry thus bridges molecular and spatial dimensions by visualizing biochemical species directly in situ. Furthermore, Wolf et al. developed TEMPO (Tracing Expression of Multiple Protein Origins), a framework integrating high-resolution proteomics of aqueous humor with single-cell transcriptomic data from all major retinal cell types. A neural network “proteomic clock” was developed to predict biological aging in specific cell types, revealing accelerated aging signals in diabetic retinopathy and uveitis even after clinical control, illustrating how AI can trace tissue-level disease processes in vivo [185]. 

While AI applications in ocular transcriptomics have expanded rapidly in recent years, several ocular tissues and diseases remain underexplored. Although substantial work has focused on the retina and the cornea, tissues like the lens, the trabecular meshwork, and the ciliary body remain comparatively underrepresented in AI-driven transcriptomic studies [21,60,148,186]. For example, while the lens has been the subject of numerous transcriptomic and multi-omics analyses, these high-resolution datasets have yet to be analyzed comprehensively using AI techniques [152,153,154,155,156,157,158,159,160,161,162,163,164].

Moving forward, multimodal machine learning frameworks may enable noninvasive inference of tissue state, immune activity, or gene regulation by integrating transcriptomic data with imaging, proteomic, or metabolomic features. In parallel, advances in spatial transcriptomics will allow molecular profiles to be mapped directly onto tissue architectures, enabling earlier detection of disease processes and finer resolution of therapeutic targets. The development of large-scale foundational models trained on diverse ocular datasets could further provide generalizable representations across diseases and modalities, reducing the need for disease-specific retraining. However, many anterior segment tissues remain largely absent from such integrative AI efforts, representing an important opportunity for future work. Achieving these advances will require robust validation using multiple datasets, alongside frameworks for interpretability, regulatory approval, and ethical deployment, ensuring that technological progress translates into tangible clinical benefits.

## Figures and Tables

**Table 1:** Summary of unsupervised machine learning algorithms used in transcriptomic analysis.

Algorithm	Modality	Biological Relevance
PCA, t-SNE, UMAP	Microarray, bulk RNA-seq, scRNA-seq	Dimensionality reduction
MAGIC	scRNA-seq	Imputes missing values/dropouts
Harmony	scRNA-seq	Corrects for batch effects
DEGreport	Microarray, bulk RNA-seq, scRNA-seq	Groups genes by correlated expression patterns
WGCNA	Microarray, bulk RNA-seq, scRNA-seq	Clusters groups of genes based on expression profiles
Leiden	scRNA-seq	Detects cell communities by clustering single-cell transcriptomes
Monocle3, Palantir	scRNA-seq	Reconstructs developmental trajectories
Seurat	Microarray, bulk RNA-seq, scRNA-seq	Comprehensive toolkit for clustering, dimensionality reduction, and batch correction
LIGER	scRNA-seq	Harmonizes data from multiple datasets
CIBERSORT, CIBERSORTx	Microarray, bulk RNA-seq	Estimates cell type composition from mixed tissue samples
GSVA	Microarray, bulk RNA-seq	Estimates variation in pathway activity across a sample population

**Table 2:** Summary of supervised machine learning algorithms used in transcriptomic analysis.

Algorithm	Category	What It Does	Strength	Limitations
LASSO	Linear model	Selects a small set of predictive genes by shrinking the contribution of less significant genes [119]	Avoids overfitting, more interpretable [120]	Assumes linear relationships; can underperform with correlated predictors [121]
SVM	Classifier	Separates classes by finding the optimal boundary [122]	Good for complex high-dimensional gene data with few samples [123]	Requires tuning
SVM-RFE	Classifier with feature elimination	Iteratively removes uninformative genes [124]	Good for complex, nonlinear, high-dimensional data [124]	Slow; does not account for correlated features [125,126]
RF	Decision tree ensemble	Combines many trees to improve accuracy and estimate gene importance [127]	Robust to noise and overfitting; more interpretable and precise [127,128]	Feature importance can be biased toward variables with more categories or more split points (e.g., continuous features) [129]
XGBoost	Gradient boosting (ensemble)	Sequentially builds decision trees to correct previous errors [130]	Handles missing values, fast and efficient [130]	Can overfit without tuning [128,131]
CatBoost	Gradient boosting (categorical)	Deals well with categorical variables, with strong generalization accuracy [128,132]	Performs well on mixed data types; minimal preprocessing of categorical features [133]	Different hyperparameters can significantly change speed/accuracy [133]
LightGBM	Gradient boosting	Uses histogram-based learning [134]	Efficient memory usage; fast training; supports large-scale data [134]	Performance may degrade on datasets with extremely high-cardinality categorical features without tuning [132]
AdaBoost	Boosted ensemble	Combines many models, correcting for mistakes made by earlier versions [135]	Fast, avoids overfitting, and handles nonlinear data well [135]	Sensitive to noise and outliers [136]
ExtraTrees	Randomized tree ensemble	Builds multiple decision trees with extra randomness to reduce overfitting [137]	Fast and avoids overfitting [137]	Less accurate and harder to interpret [137]
GRNBoost2	Tree-based with network interference	Reconstructs gene regulatory networks [138]	Captures nonlinear regulatory relationships [138]	Requires large datasets; sensitive to noise [139,140]
scGPS	Classifier [139,140] with projection scoring	Trains classifiers on labeled cell subpopulations to infer trajectories and inter-sample similarity [141]	Good for comparing single-cell populations and predicting cell fates across datasets [8]	Performance may drop with novel or underrepresented cell types [141]
xCell	Signature-based deconvolution	Estimates relative enrichment of immune cells [142]	Robust to noise, requires no retraining [142]	Limited to predefined signatures [143]

## Data Availability

No new data were created or analyzed in this study.

## Supplementary Materials

The following supporting information can be downloaded at: https://www.mdpi.com/article/10.3390/cells14171315/s1, Table S1: AI-Guided Transcriptomic Studies Across Ocular Diseases.

## Abbreviations

AI	Artificial intelligence
AMD	Age-related macular degeneration
AUCs	Areas Under the Curve
BaySeq	Bayesian Sequencing
CIBERSORT	Cell type Identification By Estimating Relative Subsets Of RNA Transcripts
DAVID	Database for Annotation, Visualization, and Integrated Discovery
DEGs	Differentially expressed genes
DESeq2	Differential Expression analysis based on the Negative Binomial distribution (version 2)
DL	Deep learning
DME	Diabetic macular edema
DNN	Deep neural networks
DR	Diabetic retinopathy
DS-NMF	Deep Subspace Nonnegative Matrix Factorization
ECM	Extracellular matrix
edgeR	Empirical Analysis of Digital Gene Expression Data in R
GO	Gene Ontology
GSEA	Gene Set Enrichment Analysis
GSVA	Gene Set Variation Analysis
GWAS	Genome-Wide Association Study Analysis
iPSC	Induced pluripotent stem cells
KCS	Keratoconjunctivitis sicca
LASSO	Least absolute shrinkage and selection operator
LIGER	Linked Inference of Genomic Experimental Relationships
LIME	Local Interpretable Model–Agnostic Explanations
limma	Linear Models for Microarray Data
MAGIC	Markov Affinity-based Graph Imputation of Cells
MCODE	Molecular Complex Detection
ML	Machine learning
NMF	Negative matrix factorization
NPDR	Non-proliferative diabetic retinopathy
OCT	Optical coherence tomography
PCA	Principal component analysis
PDMS	Polydimethylsiloxane
PDR	Proliferative diabetic retinopathy
POAG	Primary open-angle glaucoma
PVR	Proliferative vitreoretinopathy
QBAM	Quantitative brightfield absorbance microscopy
RF	Random forest
RGCs	Retinal ganglion cells
RNA-seq	RNA sequencing
RPE	Retinal pigment epithelium
SCCAF	Single-Cell Clustering Assessment Framework
ScGPS	Single-Cell Global fate Potential of Subpopulations
ScRNA-seq	Single-cell RNA sequencing
scVI	Single-cell variational inference
SHAP	SHapley Additive exPlanations
SVM	Support vector machine
TED	Thyroid eye disease
TEMPO	Tracing Expression of Multiple Protein Origins
TNF	Tumor Necrosis Factor
t-SNE	t-distributed Stochastic Neighbor Embedding
UMAP	Uniform Manifold Approximation and Projection
VAE	Variational autoencoders
VEGF	Vascular endothelial growth factor
v-SVR	Support vector regression (SVR)
WGCNA	Weighted gene co-expression network analysis
XGBoost	eXtreme Gradient Boosting
