# Supplementary material for: Artificial Intelligence in Ocular Transcriptomics: Applications of Unsupervised and Supervised Learning
Source: Cells. 2025 Aug 26;14(17):1315. doi: 10.3390/cells14171315 (PMC12427629; doi:10.3390/cells14171315)
Supplement: Supplementary file 1 [file cells-14-01315-s001.zip › cells-3808744-supplementary.pdf]

Table S1. AI-Guided Transcriptomic Studies Across Ocular Diseasess Summary of AI-guided transcriptomic studies across ocular diseases. Each study is categorized by disease, transcriptomic modality ML methods used and key findings. This table highlights how AI has been applied to uncover diagnostic biomarkers, molecular mechanisms, immune features, and regulatory programs in diverse ocular contexts.

**Author (Year)**	**Disease**	**Transcriptomic Modality**	**AI Methods**	**Key Findings**
Goetz, J. 2022	Retinal ganglion cells	scRNA-seq	XGBoost	Retinal neuronal classification
Li, R. 2023	retinogenesis	scATAC-seq scRNA-seq	SCENIC GRNBoost2 CisTarget	gene regulation in retinal development
Zhang, J. 2025	Fetal retinal development	scRNA-seq	IFS framework LASSO Ensemble ML classifiers (RF, XGBoost, CatBoost, etc.)	Gene regulation of major retinal cell types
Wang, S. 2022	Five pathologies (AMD, glaucoma, DR, myopia and type 2 macular telangiectasia)	scRNA-seq scATAC-seq	CNNs from BPNet architecture	Noncoding risk variants role in complex eye diseases
Norrie, J. 2019	Retinogenesis	scRNA-seq scATACseq	SVM Logistic Regression RF Ilastik machine learning	Gene expression during retinal development
Liang, Q. 2023	Retinal cell types	scRNA-seq scATAC-seq	ScVI	Retinal cell-type specified TF regulation
Lukowski, S. 2019	Retinal cells	scRNA-seq	scGPS	Transcriptome profile of retina
Wolf J. 2024	AMD/DMA		PCA TEMPO	Liquid biopsy proteomics

Huang, J. 2022a	DR	Bulk RNA-seq	Cytoscape<br>LASSO<br>SVM-RFE<br>WGCNA<br>GSVA	Role of Th17 cells in the progression of DME.
Huang, J. 2022b	DR	Bulk RNA-seq	Cytoscape<br>LASSO<br>SVM-RFE<br>GSVA	Role of CD8+T cells in the progression of DME.
Liu, J.2022	DR	microarray	LASSO<br>RF	DR diagnosis
Wang, J. 2022	DR	Bulk RNA-seq	Ordinal logistic regression model<br>CIBERSORT	Genes associated with severity of DR
Toh, H. 2023	DR	Bulk RNA-seq	RF	Early pathogenesis of DR
Wang, R 2022	DR	Bulk RNA-seq	GWAS	DR metabolic mechanism
Laich, Y. 2022	PVR	scRNA-seq	xCell<br>Single-cell imaging mass spectrometry	Pathophysiology of PVR
Han, D. 2023	AMD	microarray	GSVA<br>CIBERSORT<br>Supervised classifiers (LASSO, SVM, RF, XGB, GLM)	Transcriptomic biomarker for AMD for etiology research and early dignosis
Kuchroo, M. 2023	AMD	scRNA-seq	Topology-aware clustering algorithm (CATCH)	Pathophysiology of early and advanced AMD
Zhang, S. 2024	AMD	scRNA-seq<br>Bulk RNA-seq<br>microarray	GSVA<br>CIBERSORT<br>supervised classifiers (RF, AdaBoost, KNN, SVM-RFE)	AMD diagnosis
Wang, J. 2023	AMD	scRNA-seq<br>Bulk RNA-seq	CIBERSORTx<br>Ordinal logistic regression model	Relationship between age and AMD
Wang, Z. 2021	AMD	Bulk RNA-seq	CIBERSORT<br>GSVA<br>RF	Key genes for AMD pathogenesis

Oca, A. 2021	AMD	Bulk RNA-seq	RF	Predictive biomarkers for AMD treatment response
Ma, K. 2025	AMD	Bulk RNA-seq	Supervised ensemble classifiers<br>SHAP	Immune signatures of AMD
Cai, Y. 2025	keratoconus	Bulk RNA-seq<br>scRNA-seq	LASSO<br>SVM-RFE	Mitochondria-related pathologic mechanism
Cheng, Z. 2024	keratoconus	Bulk RNA-seq	CIBERSORT<br>LASSO<br>SVM-RFE<br>Naïve Bayes model	Biomarkers for keratoconus pathogenesis
Liu, J. 2024	keratoconus	Bulk RNA-seq	LASSO<br>RF	Signature genes for etiology of keratoconus
Wu, X.2023	keratoconus	microarray	SVM-RFE<br>SVM	Pathogenesis of keratoconus
Dong, Z 2024	KCS	Bulk RNA-seq	LASSO<br>SVM-RFE<br>CIBERSORT	Identified genes associated with the diagnosis and treatment of KCS
Wang, D. 2024	Glaucoma	microarray	WGCNA<br>GSVA<br>LASSO<br>RF	Diagnostic biomarkers for glaucoma
Zhao, S. 2024	POAG	microarray	LASSO<br>SVM-RFE	Biomarkers for the diagnosis of POAG
Suo, L. 2022	POAG	microarray	RF<br>SVM<br>CIBERSORT	Immune-related genes of POAG
Jia, X. 2023	POAG	scRNA-seq	t-SNE	TM structure in POAG and associated genes
Ma, K. 2025	TED	microarray	LASSO<br>SVM-RFE<br>RF<br>CIBERSORT	Signature genes for TED diagnosis
Shu, X. 2024	TED	microarray	LASSO<br>RF<br>SVM-RFE<br>XGBoost<br>CIBERSORT	Diagnostic biomarkers for TED

Lalman, C. 2025	PCO	Bulk RNA-seq	LASSO<br>RF<br>SVM-RFE	Diagnostic biomarkers of PCO
